# Zilucoplan in patients with acute hypoxic respiratory failure due to COVID-19 (ZILU-COV): A structured summary of a study protocol for a randomised controlled trial

**DOI:** 10.1186/s13063-020-04884-0

**Published:** 2020-11-19

**Authors:** Jozefien Declercq, Cedric Bosteels, Karel Van Damme, Elisabeth De Leeuw, Bastiaan Maes, Ans Vandecauter, Stefanie Vermeersch, Anja Delporte, Bénédicte Demeyere, Marnik Vuylsteke, Marianna Lalla, Trevor Smart, Laurent Detalle, René Bouw, Johannes Streffer, Thibo Degeeter, Marie Vergotte, Tanguy Guisez, Eva Van Braeckel, Catherine Van Der Straeten, Bart N. Lambrecht

**Affiliations:** 1 VIB Center for Inflammation Research, Ghent, Belgium and Department of Internal Medicine and Pediatrics, Ghent University, Ghent, Belgium; 2 University Hospital Ghent, Ghent, Belgium; 3 VIB-UGent Center for Inflammation Research, Ghent, Belgium; 4 UCB Biopharma SRL, Braine-l’Alleud, Belgium; 5 UCB Pharma, Slough, UK; 6 Ghent University, Ghent, Belgium

**Keywords:** COVID-19, Randomised controlled trial, protocol, zilucoplan, complement system, complement C5 inhibition, systemic cytokine release syndrome, cytokine storm, hypoxic respiratory failure, acute respiratory distress syndrome, ARDS

## Abstract

**Objectives:**

Zilucoplan (complement C5 inhibitor) has profound effects on inhibiting acute lung injury post COVID-19, and can promote lung repair mechanisms that lead to improvement in lung oxygenation parameters. The purpose of this study is to investigate the efficacy and safety of Zilucoplan in improving oxygenation and short- and long-term outcome of COVID-19 patients with acute hypoxic respiratory failure.

**Trial design:**

This is a phase 2 academic, prospective, 2:1 randomized, open-label, multi-center interventional study.

**Participants:**

Adult patients (≥18y old) will be recruited at specialized COVID-19 units and ICUs at 9 Belgian hospitals. The main eligibility criteria are as follows:

1) Inclusion criteria:

a. Recent (≥6 days and ≤16 days) SARS-CoV-2 infection.

b. Chest CT scan showing bilateral infiltrates within the last 2 days prior to randomisation.

c. Acute hypoxia (defined as PaO_2_/FiO_2_ below 350 mmHg or SpO2 below 93% on minimal 2 L/min supplemental oxygen).

d. Signs of cytokine release syndrome characterized by either high serum ferritin, or high D-dimers, or high LDH or deep lymphopenia or a combination of those.

2) Exclusion criteria:

e. Mechanical ventilation for more than 24 hours prior to randomisation.

f. Active bacterial or fungal infection.

g. History of meningococcal disease (due to the known high predisposition to invasive, often recurrent meningococcal infections of individuals deficient in components of the alternative and terminal complement pathways).

**Intervention and comparator:**

Patients in the experimental arm will receive daily 32,4 mg Zilucoplan subcutaneously and a daily IV infusion of 2g of the antibiotic ceftriaxone for 14 days (or until hospital discharge, whichever comes first) in addition to standard of care. These patients will receive additional prophylactic antibiotics until 14 days after the last Zilucoplan dose: hospitalized patients will receive a daily IV infusion of 2g of ceftriaxone, discharged patients will switch to daily 500 mg of oral ciprofloxacin.

The control group will receive standard of care and a daily IV infusion of 2g of ceftriaxone for 1 week (or until hospital discharge, whichever comes first), to control for the effects of antibiotics on the clinical course of COVID-19.

**Main outcomes:**

The primary endpoint is the improvement of oxygenation as measured by mean and/or median change from pre-treatment (day 1) to post-treatment (day 6 and 15 or at discharge, whichever comes first) in PaO_2_/FiO_2_ ratio, P(A-a)O_2_ gradient and a/A PO_2_ ratio.

(PAO_2_= Partial alveolar pressure of oxygen, PaO_2_=partial arterial pressure of oxygen, FiO_2_=Fraction of inspired oxygen).

**Randomisation:**

Patients will be randomized in a 2:1 ratio (Zilucoplan: control). Randomization will be done using an Interactive Web Response System (REDCap).

**Blinding (masking):**

In this open-label trial neither participants, caregivers, nor those assessing the outcomes will be blinded to group assignment.

**Numbers to be randomised (sample size):**

A total of 81 patients will be enrolled: 54 patients will be randomized to the experimental arm and 27 patients to the control arm.

**Trial Status:**

ZILU-COV protocol Version 4.0 (June 10 2020). Participant recruitment started on June 23 2020 and is ongoing. Given the uncertainty of the pandemic, it is difficult to predict the anticipated end date.

**Trial registration:**

The trial was registered on Clinical Trials.gov on May 11^th^, 2020 (ClinicalTrials.gov Identifier: NCT04382755) and on EudraCT (Identifier: 2020-002130-33).

**Full protocol:**

The full protocol is attached as an additional file, accessible from the Trials website (Additional file 1). In the interest in expediting dissemination of this material, the familiar formatting has been eliminated; this Letter serves as a summary of the key elements of the full protocol.

**Supplementary Information:**

The online version contains supplementary material available at 10.1186/s13063-020-04884-0.

## Supplementary Information

**Additional file 1.**

## Data Availability

Not applicable.

